# Evolution of Filament-Shaped Porous Structure in Polycarbonate by Stretching under Carbon Dioxide

**DOI:** 10.3390/polym10020148

**Published:** 2018-02-05

**Authors:** Tomoaki Taguchi, Tomoe Hatakeyama, Ramu Miike, Hiromu Saito

**Affiliations:** Department of Organic and Polymer Materials Chemistry, Tokyo University of Agriculture and Technology, Koganei-shi, Tokyo 184-8588, Japan; s141995z@st.go.tuat.ac.jp (T.T.); s169481v@st.go.tuat.ac.jp (T.H.); s150622w@st.go.tuat.ac.jp (R.M.)

**Keywords:** polycarbonate, porous structure, carbon dioxide, void, stretching, plasticization, hydrostatic pressure

## Abstract

We found that a filament-shaped porous structure with periodic distance was obtained in polycarbonate for optical disk grade (OD-PC) film by stretching under compressed carbon dioxide (CO_2_). The evolution of the characteristic porous structure was investigated by in situ observation during the stretching under compressed CO_2_ and the optical microscopic observation of the stretched specimen. The voids were obtained under high CO_2_ pressure as in the case of elevated temperature, suggesting that the evolution of the voids was caused by crazing due to chain disentanglement by accelerated molecular motion owing to the plasticization effect of CO_2_. The filament-shaped voids were initiated at around the yielding point and increased continuously by nucleation in the matrix around the surface of the pre-existing voids. The shape of the voids did not change to an ellipsoidal one during stretching due to suppression of the craze opening by the hydrostatic pressure effect. The stretching of the CO_2_-absorbed depressurized OD-PC revealed that the initiation of the voids was not only caused by the plasticization effect, but the hydrostatic pressure effect was also required.

## 1. Introduction

It is well known that carbon dioxide (CO_2_) is nontoxic and compressed CO_2_ can be absorbed into polymers. By absorbing CO_2_ into polymers, polymers are plasticized. Owing to the plasticization by CO_2_, chain mobility is accelerated [1,2,3,4,5], deformation is enhanced [6,7,8,9,10], the glass transition temperature is depressed [11,12,13,14,15], and viscosity decreases [15,16,17,18,19] with increase of CO_2_ pressure, subsequently dissolving the amount in the polymer. Our recent study on the stress–strain behavior of poly(methyl methacrylate) under compressed CO_2_ revealed that molecular orientation was enhanced and the elongation at break increased with the increased pressure of CO_2_ due to the plasticization effect when the amount of gas absorbed into the polymers was large at high CO_2_ pressure [20]. On the other hand, the elongation at break was found to be decreased with increased pressure of CO_2_ in low molecular weight polycarbonate for optical disc grade (OD-PC) [21]. In addition to the plasticization effect, compressed CO_2_ caused the hydrostatic pressure effect, which is the resistance to deformation caused by the vitrification resulting from the reduction of free volume.

One application of the plasticization by compressed CO_2_ is in the production of porous materials. Porous materials made of polymers are used in a wide range of commercial applications because of their properties of low density, high impact resistance, low dielectric constant, and good thermal insulation [22]. A well-known method of producing porous materials by using CO_2_ is foaming [23,24,25,26,27,28,29,30,31,32,33]. When the polymer/CO_2_ mixture is quenched into a supersaturated state by depressurization or heating, a large number of gas bubbles nucleate spontaneously and grow with spherical symmetry, resulting in a closed-cell material as spherical bubbles are entrapped within a continuous polymer phase. On the other hand, open cellular materials with a highly interconnected porous structure can be developed by liquid–liquid phase separation via spinodal decomposition of the polymer/CO_2_ mixture [34,35]. Nanoporous materials with various structures and sizes were obtained by drying solvent from the gels using supercritical CO_2_ without collapse of the structure [36,37], and a fine-layered porous structure was obtained by the growth of bubbles in the constrained intercrystalline amorphous region [38,39].

Porous materials can also be obtained by stretching under compressed CO_2_. The porous structure obtained by stretching is usually caused by crazing. By using this method, one can obtain a porous structure with various sizes and shapes by controlling the crazing. A craze consists of a dense array of fibrils separated by voids. There are two distinct mechanisms responsible for crazing [40,41,42,43,44,45]. One mechanism is scission crazing, which is caused by the breaking of molecular chains during fibril formation. Another is disentanglement crazing, which is caused by chain disentanglement due to accelerated molecular motion. In crystalline polymers, voids are formed in the intercrystalline amorphous region by stretching [46] and the void formation is enhanced by stretching under CO_2_ [10,47]. On the other hand, in amorphous polymers, we found that a filament-shaped porous structure with periodic distance was obtained macroscopically throughout the whole specimen in OD-PC by stretching under compressed CO_2_ [21]. Such a filament-shaped porous structure was not obtained by conventional stretching under air at atmospheric pressure, but by a specially designed continuous three-point bending method [48,49,50]. Several applications have been suggested for a filament-shaped porous structure; e.g., privacy protector film (transparent from the front view while opaque from a diagonal direction [48]), and piece dyeing [49].

In this paper, to clarify the plasticization effect of CO_2_ on the evolution of the characteristic filament-shaped porous structure of the OD-PC obtained under compressed CO_2_, we carried out the optical microscopic observation of the stretched specimen obtained under compressed CO_2_, and the results were discussed through a comparison with those obtained under elevated temperatures at atmospheric pressure. To understand the origin of the filament-shaped porous structure, we investigated the evolution of the porous structure by in situ observation and stress–strain behavior during the stretching under compressed CO_2_. The result for the stretching of the CO_2_-absorbed depressurized specimen and that under compressed nitrogen (N_2_) are also presented to clarify the plasticization effect and that of the hydrostatic pressure.

## 2. Materials and Methods

The specimen used in this study was a commercial low molecular weight polycarbonate for optical disc grade (OD-PC) supplied by the Mitsubishi Gas Chemical Company, Inc (Tokyo, Japan) (Iupilon H-4000, *M*_v_ = 1.5 × 10^4^ g/mol). The polycarbonate pellets were compression molded between metal plates at 200 °C for 5 min to obtain film specimens with a thickness of about 200 µm, which were then gradually cooled to room temperature.

**Figure 1:**
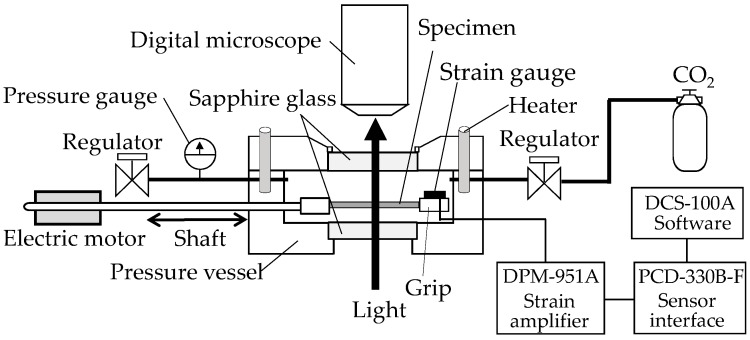
Schematic illustration of a stretching instrument for tensile-deformation measurements and in situ observation under high-pressure CO_2_.

In situ observation of the structural change of the film specimens during the stretching under compressed CO_2_ was performed using a specially designed stretching instrument with a stainless steel pressure vessel (Figure 1), as mentioned in the details of our previous papers [20,21]. The structure was observed under a high-fidelity digital microscope (MX-5030SZII, and KH-1300, Hirox Co., Ltd., Tokyo, Japan) equipped above a pressure vessel through a sapphire window. The crosshead of the stretching instrument traveled to a strain limit of 2.8 in the pressure vessel (Taiatsu Techno Corporation, Tokyo, Japan). Movement of the crosshead was regulated by a shaft connected to a linear motor (LD1208-244, Oriental Motor Co., Ltd., Tokyo, Japan) outside the vessel. A strain gauge (KFR-2-120-C1-16, Kyowa Electronic Instruments Co., Ltd., Tokyo, Japan) was attached to the surface of the crosshead to measure the stress during the stretching of the OD-PC film. The voltage caused by the deformation of the strain gauge was recorded, then the applied stress was acquired with DCS-100A software (Kyowa Electronic Instruments Co., Ltd., Tokyo, Japan).

A dumbbell-shaped film specimen was cut from the OD-PC film and was mounted on the stretching instrument. After sealing, high-pressure CO_2_ was injected into the pressure vessel with a syringe pump (NPKX-500, Nihon Seimitsu Kagaku Co., Ltd., Tokyo, Japan) at room temperature and maintained for 1 h to dissolve any CO_2_ in the specimen. Next, the specimen was stretched at a stretching speed of 0.05 s^−1^ under CO_2_. It was noted that the tensile properties of the OD-PC films did not change by absorbing CO_2_ for longer than 1 h, so 1 h was sufficient for absorbing the CO_2_ in the specimen [21]. In situ observation under CO_2_ was carried out at a stretching speed of 8.33 × 10^−2^ s^−1^ where the porous structure obtained was almost the same as that obtained by stretching at a stretching speed of 0.05 s^−1^, to obtain the structure image during the measurement of the stress–strain behavior at small strain. The pressure of CO_2_ within the vessel was monitored with an output pressure transducer and kept constant with a back-pressure regulator (TESCOM^TM^ 26-1763-24, Emerson Electric Co., St. Louis, MO, USA). The temperature was set at 30 °C by an autotune temperature controller unit with a thermocouple for measurement under CO_2_, and was set at a suitable temperature for measurement under air at atmospheric pressure. To clarify the hydrostatic pressure effect, the stress–strain behavior was also measured under compressed N_2_ for a short period within 5 min after injection of N_2_ into the pressure vessel to eliminate absorption into the specimen.

The structure of the stretched specimen was observed after depressurization of CO_2_ using an optical microscope (BX53-P, Olympus Corporation, Tokyo, Japan) equipped with a charge-coupled device (CCD) camera (DP73, Olympus Corporation, Tokyo, Japan). The morphology under polarized optical microscopy was observed through the optical microscope equipped with a sensitive tint plate with an optical path difference of 530 nm under crossed polarizers. Each stretched specimen was also observed under a scanning electron microscope (SEM) (S2100A, Hitachi High-Technologies Corporation, Tokyo, Japan). For observation, the surface of the specimen was sputter-coated with platinum.

## 3. Results and Discussion

**Figure 2:**
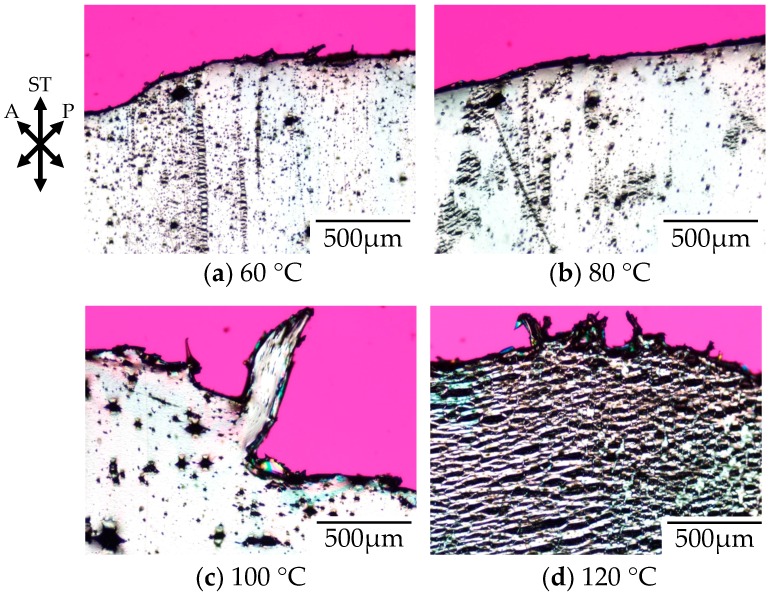
Polarized optical micrographs of stretched-and-fractured low molecular weight polycarbonate for optical disc grade (OD-PC) obtained by stretching under atmospheric pressure at various temperatures.

Figure 2 shows the polarized optical micrographs of stretched-and-fractured polycarbonate obtained by stretching under atmospheric pressure at various temperatures. Here, the polycarbonate used in this study was a low molecular weight for optical disc grade (OD-PC). As demonstrated in our previous paper, the tensile property of the OD-PC was ductile at room temperature and changed from ductile to brittle through elevated temperatures [21]. No void was obtained at room temperature where the tensile property was ductile. At 60 °C, the tensile property became brittle and small voids with a size of several micrometers appeared (Figure 2a). Large voids with a size of several tens of micrometers were obtained at higher temperatures above 60 °C, and the number of large voids increased with increasing temperature (Figure 2b,c). At 120 °C, large ellipsoidal lace-like porous structures with sizes of several tens of micrometers were obtained (Figure 2d). Such porous structures were induced by crazing. Since crazing occurred at elevated temperatures and the size of the voids increased with increasing temperature, the crazing was caused by intermolecular separation due to chain disentanglement by accelerated molecular motion owing to the elevated temperature [40,41,42,43,44,45].

**Figure 3:**
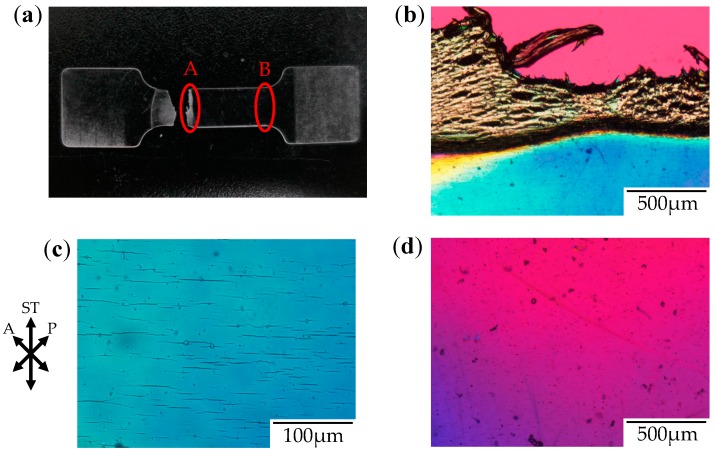
Photograph and polarized optical micrographs of OD-PC obtained by stretching under atmospheric pressure at 120 °C. (**a**) Photograph of the fractured specimen; (**b**) Polarized optical micrograph for region A; (**c**) Magnification of the lower part of (**b**); and (**d**) Polarized optical micrograph for region B.

Figure 3 shows the photograph and the polarized optical micrographs at different positions of the stretched-and-fractured OD-PC obtained by stretching under atmospheric pressure at 120 °C. At region A in Figure 3a, large ellipsoidal voids were seen around the edge of the fractured surface (Figure 3b), while short and fine line-shaped voids were observed at the outer region, several hundred micrometers from the edge of the fractured surface (Figure 3c). These results suggest that short and fine line-shaped crazes changed to ellipsoidal ones by craze openings in the stretching direction, so large lace-like porous structures like those shown in Figure 2d were obtained. We noted that the interference color around the large ellipsoidal voids was quite different from that around the line-shaped ones as the interference color is related to retardation (birefringence), i.e., it changes from red–purple, blue, green to white with increasing retardation. Since the retardation is related to molecular orientation, the difference of the interference color for the short line-shaped voids and large ellipsoidal ones might be attributed to the different molecular orientation for crazing. At region B in Figure 3a, no structure or retardation were seen (Figure 3d), suggesting that the molecular orientation was small so that craze was not induced. Thus, the porous structure was obtained only in a narrow region due to local orientation in the specimen by stretching under air at atmospheric pressure. When the porous structure is formed, the specimen becomes white due to the scattering of light caused by the reflective index difference between the OD-PC matrix and air in the void. Owing to the porous structure obtained only at a narrow region, the stretched-and-fractured OD-PC was white only around the edge of the fractured surface (Figure 3a).

**Figure 4:**
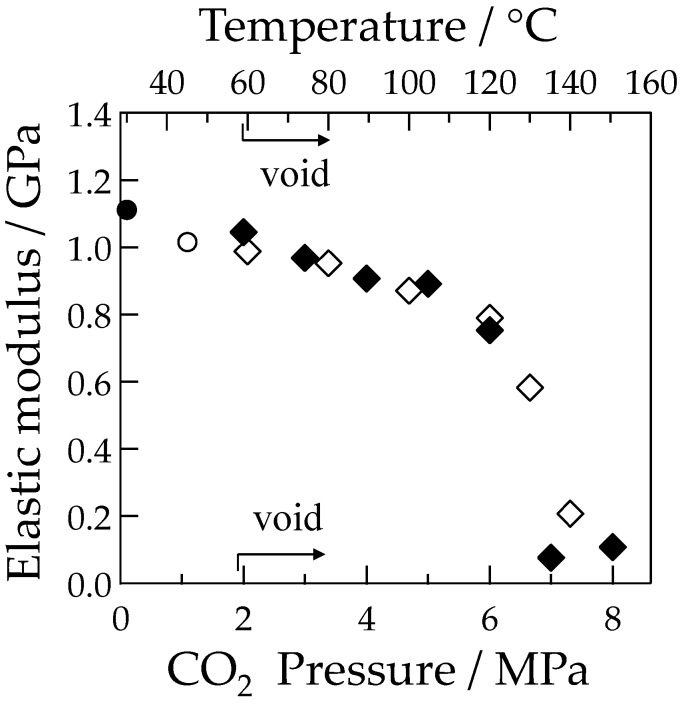
Elastic modulus of the OD-PC as a function of temperature and CO_2_ pressure; ○♢, temperature; ●♦, CO_2_ pressure; ○●, without void; ♢♦, void.

As shown in Figure 4, the elastic modulus, obtained from the initial slope in the elastic region of the stress–strain curve, gradually decreased with increasing temperature due to accelerated molecular motion, then decreased sharply at temperatures above 120 °C by approaching the glass transition temperature [21]. Voids were obtained by stretching at a temperature above 60 °C as indicated by the rhombus symbols shown in Figure 4.

**Figure 5:**
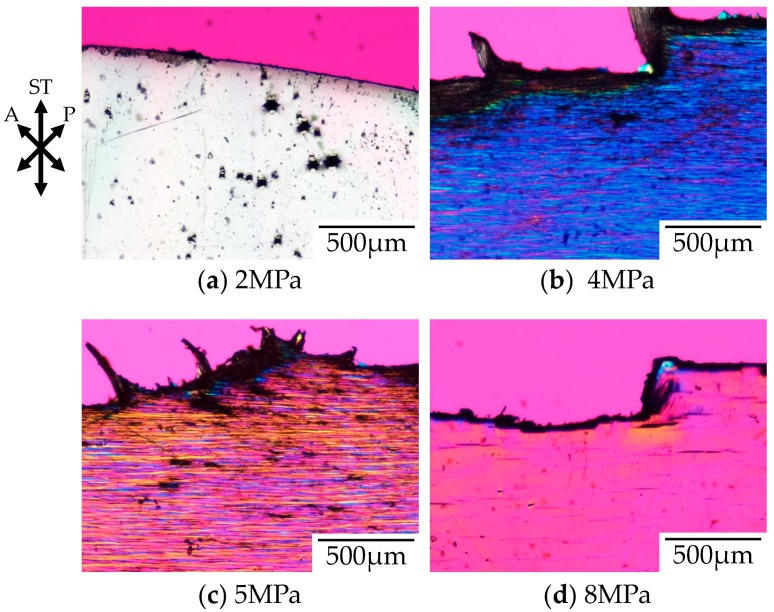
Polarized optical micrographs of stretched-and-fractured OD-PC obtained by stretching at 30 °C under CO_2_ of various pressures.

Figure 5 shows the polarized optical micrographs of the stretched-and-fractured OD-PC obtained by stretching at 30 °C under CO_2_ of various pressures. As observed under elevated temperatures, the tensile property changed from ductile to brittle with increases to the CO_2_ pressure [21]. No void was obtained under air at atmospheric pressure where the tensile property was ductile. At 2 MPa under CO_2_, the tensile property became brittle and small numbers of distorted-shaped voids with a size of several tens of micrometers were obtained (Figure 5a). Such ductile-to-brittle transition with increasing CO_2_ pressure was the opposite to the brittle-to-ductile transition observed in poly(methyl methacrylate) [20]. As shown in Figure 4, the elastic modulus decreased with increasing CO_2_ pressure due to accelerated molecular motion as in the case of elevating temperatures. Though the elastic modulus under CO_2_ at 2 MPa was the same as that under atmospheric pressure at 60 °C, the porous structure obtained under CO_2_ at 2 MPa was different from that under atmospheric pressure at 60 °C (Figure 2a), but was almost the same as that under atmospheric pressure at 100 °C (Figure 2c). On the other hand, a filament-shaped porous structure with periodic distance was obtained under CO_2_ at 4 and 5 MPa (Figure 5b,c). The void was long and perpendicular to the stretching direction, i.e., the length was more than several hundred micrometers while the width in the stretching direction was several micrometers. This porous structure was quite different from that obtained under CO_2_ at 2 MPa and under atmospheric pressure at elevated temperatures. A fibrillar-shaped porous structure was not obtained by stretching crystalline polymers under CO_2_ where small voids (in a nanometer scale) were formed in the intercrystalline amorphous region [10,46,47], but this structure was similar to that obtained by a specially designed continuous three-point bending method [48,49,50].

Since the voids appeared under CO_2_ above 2 MPa and the number of voids increased with increasing CO_2_ pressure, the voids were induced by crazing due to chain disentanglement by accelerated molecular motion with increasing CO_2_ pressure, as in the case of the elevated temperatures shown in Figure 2. As the elastic modulus decreased with the increased amount of absorbed CO_2_ by associating with the increased CO_2_ pressure (Figure 4), the accelerated molecular motion was attributed to the plasticization effect of CO_2_. The elastic modulus decreased sharply at a CO_2_ pressure above 6 MPa because the measuring temperature under CO_2_ approached the glass transition temperature by depression of the glass transition temperature with increased CO_2_ pressure. Though the number of voids increased with increasing CO_2_ pressure below 5 MPa, it decreased drastically with increasing CO_2_ pressure above 5 MPa and small numbers of voids were formed under CO_2_ at 8 MPa (Figure 5d). The drastic decrease of the number of voids at CO_2_ pressure above 5 MPa might be attributed to the fracture of the specimen before the increase of the number of crazes because the strain at break decreased drastically with increasing CO_2_ pressure above 5 MPa due to the large increase of the chain disentanglement as the stretching temperature under CO_2_ was close to the glass transition temperature as inferred from Figure 4. The interference color obtained under CO_2_ of 8 MPa was red–purple, which was the same as that outside the specimen, suggesting a small orientation due to large chain disentanglement during stretching by a large plasticization effect at high CO_2_ pressure above 5 MPa.

**Figure 6:**
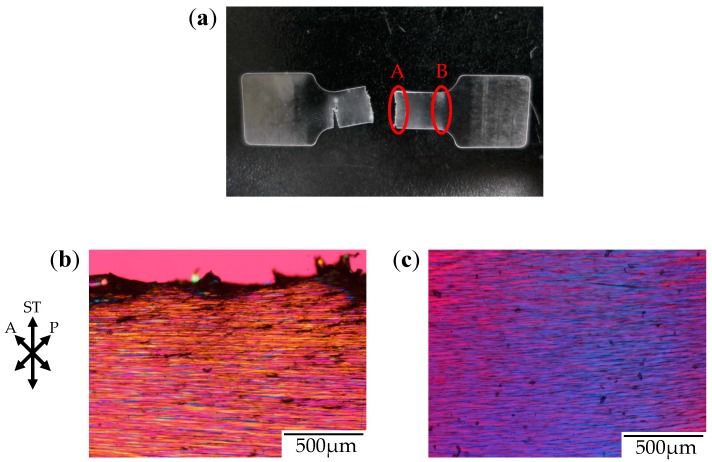
Photograph and polarized optical micrographs of OD-PC obtained by stretching under CO_2_ at 5 MPa. (**a**) Photograph of the fractured specimen; (**b**) Polarized optical micrograph for region A; and (**c**) Polarized optical micrograph for region B.

Figure 6 shows the photograph and the polarized optical micrographs at different positions of the stretched-and-fractured OD-PC obtained by stretching under CO_2_ at 5 MPa. Filament-shaped voids were seen as long black lines surrounded by a long fibrillar matrix with interference colors of yellow and red–purple (Figure 6b,c). A periodic structure with different interference colors was observed along the filament-shaped voids, indicating that the porous structure developed uniformly with periodic distance. The blue interference color in the matrix indicates that the OD-PC molecules in the fibrillar matrix were oriented in the stretching direction, while the red–purple color indicated no orientation. Hence, the different interference colors in the matrix suggested the distribution of the molecular orientation. The distribution of the molecular orientation might be attributed to the preferential orientation of the OD-PC matrix around the surface of the voids due to stress concentration induced during the void formation. The difference of the porous structure at regions A and B in Figure 6a was small. Thus, the porous structure was uniformly obtained in the specimen at a wide region by stretching under compressed CO_2_, while the porous structure was only obtained locally around the fractured edge under atmospheric pressure at elevated temperatures (Figure 3). Given the porous structure obtained at a wide region, the stretched-and-fractured specimen was white at the wide region (Figure 6a).

**Figure 7:**
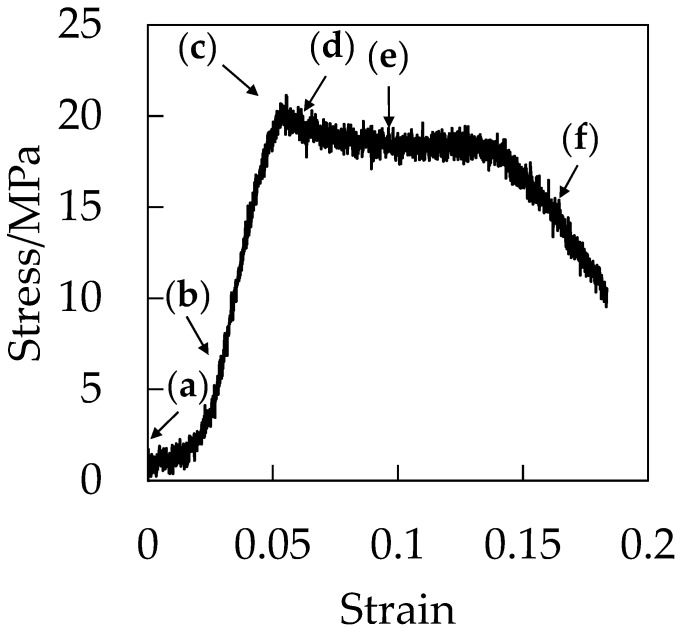
Stress–strain curve of OD-PC during stretching under CO_2_ at 5 MPa.

To understand the origin of the evolution of the characteristic filament-shaped porous structure shown in Figure 5 and Figure 6, we carried out stress–strain measurements and in situ observations during the stretching under CO_2_. Figure 7 shows the stress–strain curve during the stretching under CO_2_ at 5 MPa. Initially, the stress increased in a linear manner with increased strain at the elastic region before the stress reached a maximum at the yielding point around the strain of 0.06. After the yielding point, the plasticized plateau region was observed and the specimen was elongated to a strain up to 0.18 at the plastic deformation region. Fracture at the small strain of 0.18 is characteristic of brittle behavior. As demonstrated in our previous paper, the elongation at break became shorter as CO_2_ pressure increased [21].

**Figure 8:**
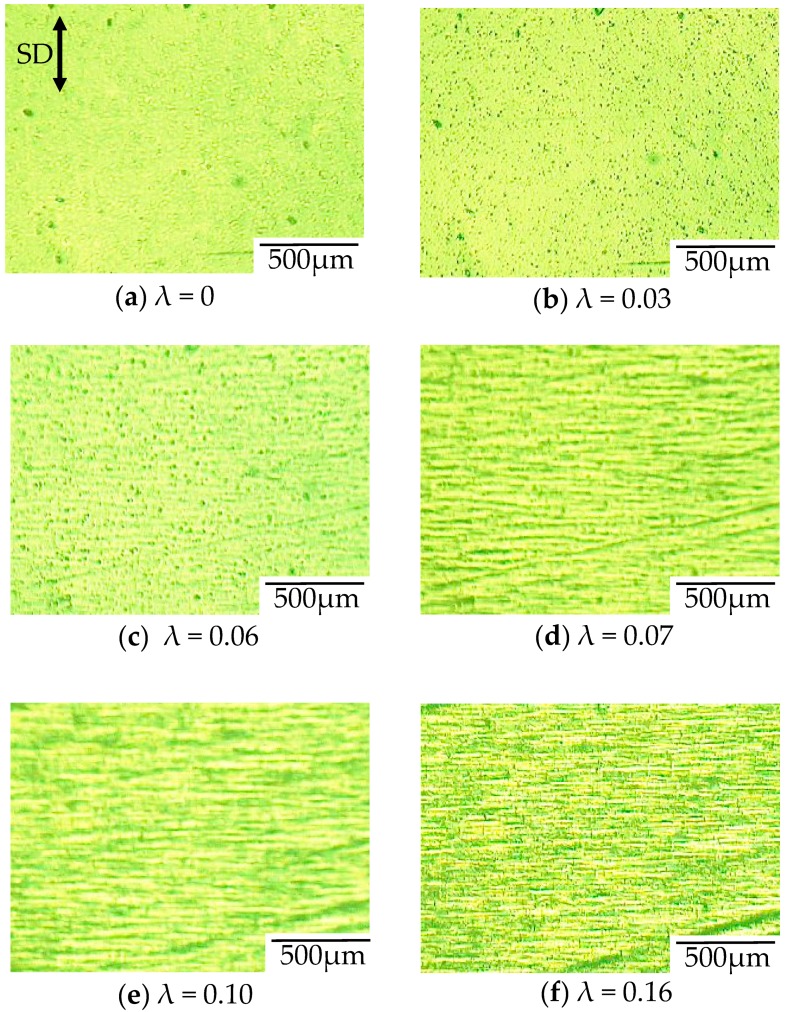
In situ observation for void formation of OD-PC during stretching under CO_2_ at 5 MPa.

Figure 8 shows the optical micrographs of the in situ observation for the void formation of OD-PC during the stretching under CO_2_ at 5 MPa for the points (a–f) in the stress–strain curve of Figure 7. Before stretching, a small contrast was seen. This contrast was not caused by void formation, but by the reflection of light from the surface of the specimen due to the small roughness of the specimen (Figure 8a). No structural change was seen at the elastic region before the yielding point (Figure 8a,b). Filament-shaped voids appeared in the OD-PC matrix around the yielding point (Figure 8c,d), suggesting that crazing for the filament-shaped voids was initiated around the yielding point. The results indicated that the voids observed in Figure 5 and Figure 6 were not obtained by foaming [23,24,25,26,27,28,29,30,31,32,33] during the depressurization after stretching, but by crazing during the stretching. Hence, the porous structure obtained was quite different from the spherical-shaped one obtained through foaming. The thickness of the voids became larger without changing its shape, and the number of voids increased continuously during the stretching at the plastic deformation region (Figure 8c,f). The number of voids increased by nucleation in the matrix between the pre-existing ones, and then evolved to the filament-shaped porous structure with periodic distance. Though the line-shaped void changed to an ellipsoidal one through the craze opening under atmospheric pressure at elevated temperatures, the filament-shaped void did not change to an ellipsoidal one under compressed CO_2_, suggesting the suppression of the craze opening under compressed CO_2_. Thus, the filament-shaped porous structure developed under CO_2_ might be obtained by the increase of the number of filament-shaped voids uniformly in the matrix and the suppression of the craze opening.

**Figure 9:**
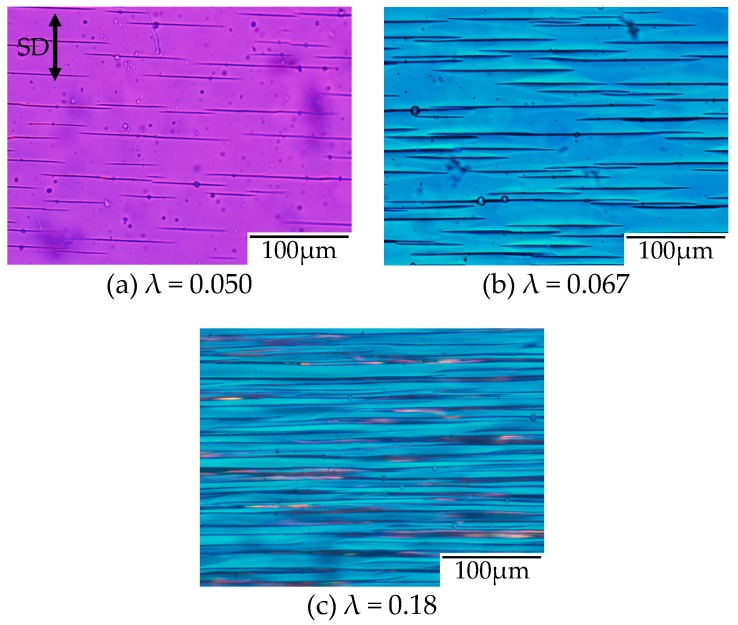
Polarized optical micrographs of OD-PC obtained by stretching under CO_2_ at 5 MPa at various strains.

To confirm the evolution of the filament-shaped porous structure suggested by the in situ observation shown in Figure 8, the polarized optical micrographs for the porous structure obtained after stretching at various strains under CO_2_ of 5 MPa are shown in Figure 9. A small number of long filament-shaped voids were obtained in the OD-PC matrix around the yielding point (Figure 9a). The interference color around the surface of the voids was different from that at the outer region. The difference was attributed to the large orientation due to the stress concentration around the surface of the voids induced during the void formation. The interesting result here was that new voids were developed around the surface of the pre-existing voids (Figure 9b). This suggested that the crazing occurred preferentially around the surface of the pre-existing voids where the molecular orientation was large. Such behavior may be the same as the extensional stress effect suggested in the foaming [31,32]. The number of voids increased continuously in the matrix with increased strain and evolved to the filament-shaped porous structure with periodic distance (Figure 9c). The filament-shaped voids became longer in the perpendicular direction of the stretching one. On the other hand, the increase of the void thickness was small, so the long filament-shaped voids did not change to ellipsoidal ones due to the suppression of the craze opening. These results support the evolution of the filament-shaped porous structure suggested by the in situ observation during the stretching under CO_2_ shown in Figure 8.

**Figure 10:**
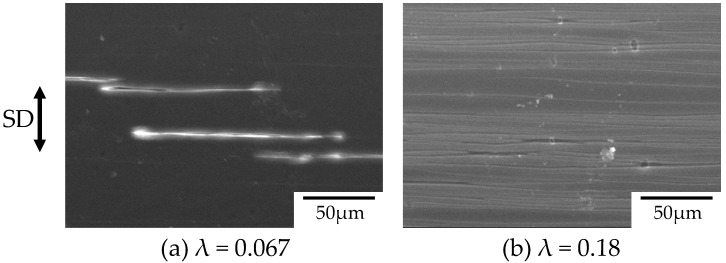
Scanning electron microscope (SEM) micrographs of the surface of the stretched OD-PC obtained by stretching under CO_2_ at 5 MPa at strains corresponding to Figure 9b,c.

Figure 10 shows the SEM micrographs of the surface of the stretched OD-PC obtained after stretching at various strains under CO_2_ of 5 MPa corresponding to the polarized optical micrographs shown in Figure 9b,c. Long filament-shaped voids were obtained at the surface by stretching at λ = 0.067 (Figure 10a). Short-and-small voids existed around the surface of the long-large voids. These results confirmed that the new voids developed around the surface of the pre-existing voids suggested by Figure 9b. The filament-shaped voids became longer with an increased number of voids (Figure 10b), as suggested by Figure 9c.

**Figure 11:**
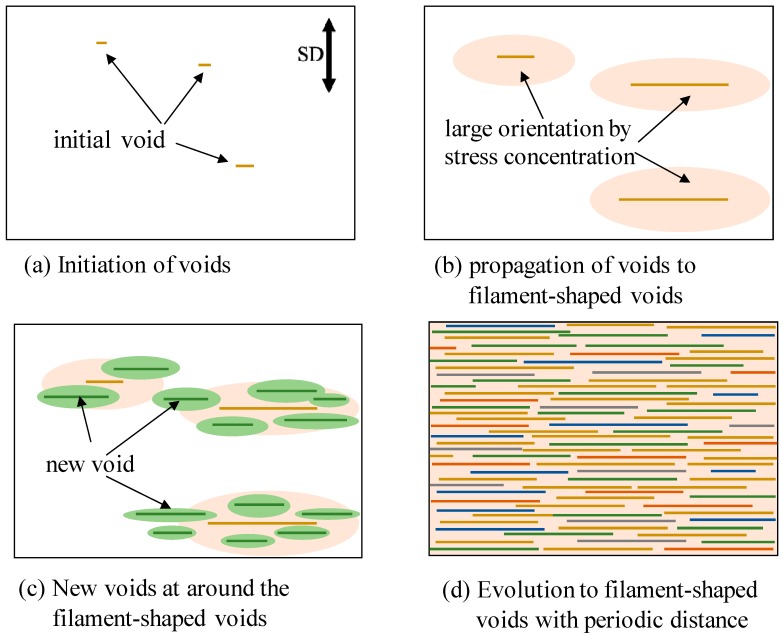
Schematic illustration for the evolution of a filament-shaped porous structure of OD-PC by stretching under CO_2_.

Figure 11 shows the schematic illustration for the evolution of the filament-shaped porous structure under compressed CO_2_. When the OD-PC film was stretched, initial voids formed in the OD-PC matrix around the yielding point (Figure 11a). The voids propagated in the perpendicular direction to the stretching one during the stretching. Due to the propagation of the voids, molecular orientation around the surface of the voids became larger than that at the outer region by stress concentration (Figure 11b). New voids developed preferentially around the surface of the voids where the molecular orientation was large through stress concentration (Figure 11c). When new voids nucleated around the surface of the voids before the growth of voids in the longitudinal direction, large numbers of voids developed before fracture. By increasing the number of the filament-shaped voids continuously without changing their shape due to the suppression of the craze opening, a filament-shaped porous structure with periodic distance was obtained (Figure 11d). Dilatational stress was required for the craze opening [51,52]. Since hydrostatic pressure is present under compressed CO_2_, the dilatational stress required for craze opening should be larger than that under atmospheric pressure, so the craze opening is suppressed under compressed CO_2_.

**Figure 12:**
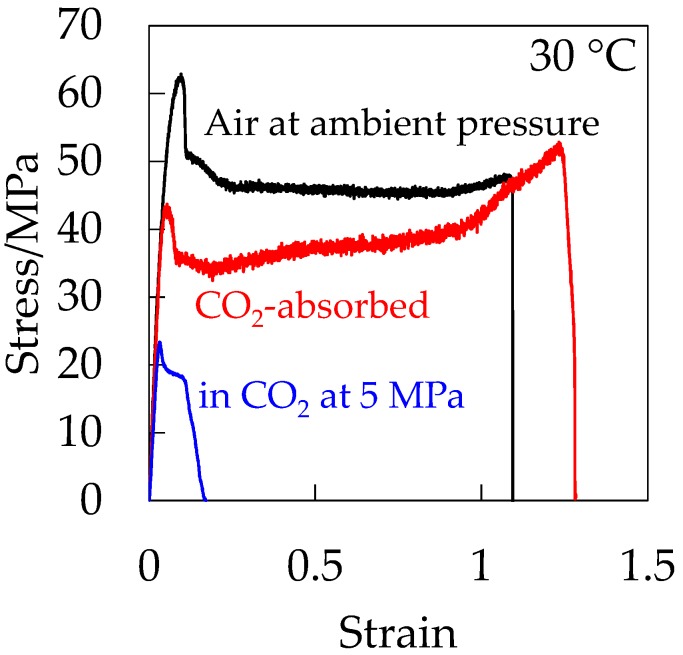
Stress–strain curve of OD-PC under air at atmospheric pressure in CO_2_ at 5 MPa and CO_2_-absorbed PC under air at atmospheric pressure after absorbing and releasing CO_2_ of 5 MPa.

To clarify the plasticization effect on the initiation of a porous structure by excluding the hydrostatic pressure effect under compressed CO_2_, the stress–strain behavior of CO_2_-absorbed depressurized OD-PC was measured under air at atmospheric pressure after absorbing and releasing CO_2_ at 5 MPa. Figure 12 shows the stress–strain curve of the CO_2_-absorbed depressurized OD-PC in the absence of hydrostatic pressure. For comparison, the stress–strain curves of OD-PC under CO_2_ at 5 MPa and under air at atmospheric pressure are also shown in Figure 12. The CO_2_-absorbed depressurized OD-PC exhibited ductile behavior, i.e., the elongation at break was much longer in the absence of hydrostatic pressure than those under compressed CO_2_ at 5 MPa and under air at atmospheric pressure. These results indicated that the property became ductile through the plasticization effect due to absorbed CO_2_ in the absence of hydrostatic pressure, although it became brittle in the presence of hydrostatic pressure under compressed CO_2_ at 5 MPa. Thus, the decrease of deformability under CO_2_ was attributed to the hydrostatic pressure effect under compressed CO_2_.

**Figure 13:**
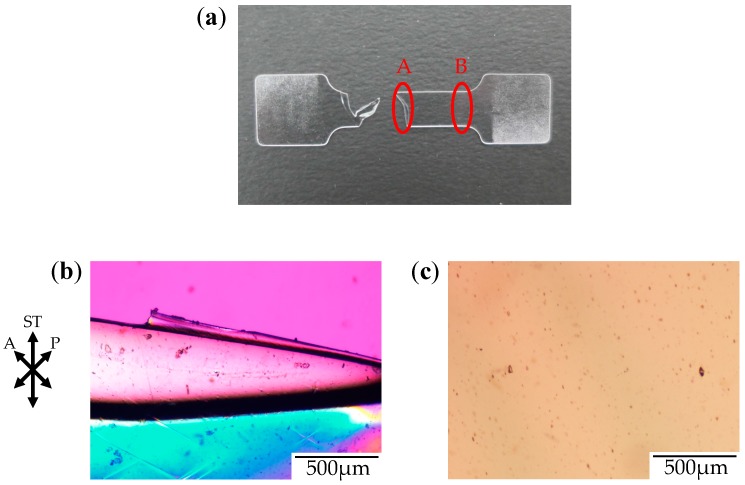
Photograph and polarized optical micrographs of OD-PC obtained by stretching CO_2_-absorbed PC under air at atmospheric pressure at 30 °C after absorbing and releasing CO_2_ at 5 MPa: (**a**) Photograph of fractured specimen; (**b**) Polarized optical micrograph for region A; and (**c**) Polarized optical micrograph for region B.

Figure 13 shows the photograph of the stretched-and-fractured CO_2_-absorbed depressurized OD-PC and the polarized optical micrographs at different positions obtained by stretching under air at atmospheric pressure after absorbing and releasing CO_2_ at 5 MPa. No voids were seen in the absence of hydrostatic pressure, although a filament-shaped porous structure was obtained in the presence of hydrostatic pressure under compressed CO_2_ as demonstrated previously. These results indicated that the voids were not only initiated by the accelerated molecular motion owing to the plasticization effect of CO_2_ in the absence of hydrostatic pressure, but were initiated in the presence of hydrostatic pressure under compressed CO_2_ at 5 MPa. Thus, the filament-shaped structure was not only obtained by the plasticization effect of CO_2_, but the hydrostatic effect was also required for the initiation of the porous structure induced by crazing.

**Figure 14:**
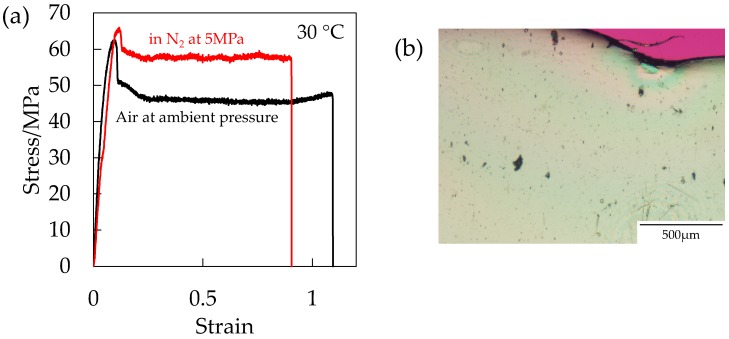
(**a**) Stress–strain curve of OD-PC under N_2_ at 5 MPa and under air at atmospheric pressure; (**b**) polarized optical micrographs of OD-PC obtained by stretching under N_2_ at 30 °C.

To clarify the hydrostatic pressure effect on the initiation of the porous structure by excluding the plasticization effect, the stress–strain behavior was measured under compressed N_2_ given that the solubility of N_2_ in polymers is much smaller than that of CO_2_ [20]. The measurement was carried out at a short period within 5 min after the injection of N_2_ into the pressure vessel to eliminate the absorption into the specimen. Figure 14a shows the stress–strain curve of the OD-PC under N_2_ at 5 MPa in the absence of the plasticization effect and that under air at ambient pressure. Strain at break was shorter under compressed N_2_ than under air at ambient pressure, suggesting that the deformability decreased by the hydrostatic pressure effect. Though the deformability decreased, voids did not appear (Figure 14b). By combining the results of Figure 12 and Figure 13, the results indicated that both the hydrostatic pressure effect and the plasticization effect of CO_2_ were required for the initiation of the porous structure. Thus, the filament-shaped porous structure obtained under compressed CO_2_ might be attributed to both the extensional stress, which is effective for void formation [31,32] induced by the hydrostatic pressure effect and the disentanglement due to accelerated molecular motion by plasticization effect.

## 4. Conclusions

A filament-shaped porous structure with periodic distance was found to be obtained uniformly at a wide region in low molecular weight polycarbonate for optical disk grade (OD-PC) film by stretching under compressed CO_2_ at a high pressure around 5 MPa, while a large ellipsoidal lace-like porous structure was obtained locally around the fractured surface under atmospheric pressure at high temperatures around 120 °C. The void was obtained under high CO_2_ pressure in the case of elevated temperature, suggesting that the evolution of the voids was caused by crazing due to chain disentanglement by accelerated molecular motion owing to the plasticization effect of CO_2_. The in situ observation and stress–strain measurement during the stretching under CO_2_ revealed that filament-shaped voids were initiated around the yielding point and increased during the stretching by nucleation around the surface of the pre-existing voids where the molecular orientation was large due to the stress concentration induced by the void formation. The evolution of the filament-shaped porous structure without any change in shape despite the stretching was attributed to the suppression of the craze opening by the hydrostatic pressure effect. The initiation of the voids was not only caused by the plasticization effect of CO_2_, but the hydrostatic pressure effect was also required. The filament-shaped porous structure obtained under compressed CO_2_ might be attributed to the extensional stress induced by the hydrostatic pressure effect and the disentanglement due to accelerated molecular motion by plasticization effect.
